# Insights Into Protein *S*-Palmitoylation in Synaptic Plasticity and Neurological Disorders: Potential and Limitations of Methods for Detection and Analysis

**DOI:** 10.3389/fnmol.2018.00175

**Published:** 2018-05-29

**Authors:** Monika Zaręba-Kozioł, Izabela Figiel, Anna Bartkowiak-Kaczmarek, Jakub Włodarczyk

**Affiliations:** Laboratory of Cell Biophysics, Department of Molecular and Cellular Neurobiology, Nencki Institute of Experimental Biology, Polish Academy of Sciences, Warsaw, Poland

**Keywords:** *S*-palmitoylation, synapse, neurodegenerative diseases, metabolic labeling, biochemical methods, synaptic plasticity

## Abstract

*S*-palmitoylation (S-PALM) is a lipid modification that involves the linkage of a fatty acid chain to cysteine residues of the substrate protein. This common posttranslational modification (PTM) is unique among other lipid modifications because of its reversibility. Hence, like phosphorylation or ubiquitination, it can act as a switch that modulates various important physiological pathways within the cell. Numerous studies revealed that S-PALM plays a crucial role in protein trafficking and function throughout the nervous system. Notably, the dynamic turnover of palmitate on proteins at the synapse may provide a key mechanism for rapidly changing synaptic strength. Indeed, palmitate cycling on postsynaptic density-95 (PSD-95), the major postsynaptic density protein at excitatory synapses, regulates the number of synaptic α-amino-3-hydroxy-5-methyl-4-isoxazolepropionic acid receptors (AMPARs) and thus affects synaptic transmission. Accumulating evidence suggests a relationship between impairments in S-PALM and severe neurological disorders. Therefore, determining the precise levels of S-PALM may be essential for understanding the ways in which this PTM is regulated in the brain and controls synaptic dynamics. Protein S-PALM can be characterized using metabolic labeling methods and biochemical tools. Both approaches are discussed herein in the context of specific methods and their advantages and disadvantages. This review clearly shows progress in the field, which has led to the development of new, more sensitive techniques that enable the detection of palmitoylated proteins and allow predictions of potential palmitate binding sites. Unfortunately, one significant limitation of these approaches continues to be the inability to use them in living cells.

## Introduction

**FIGURE 1:**
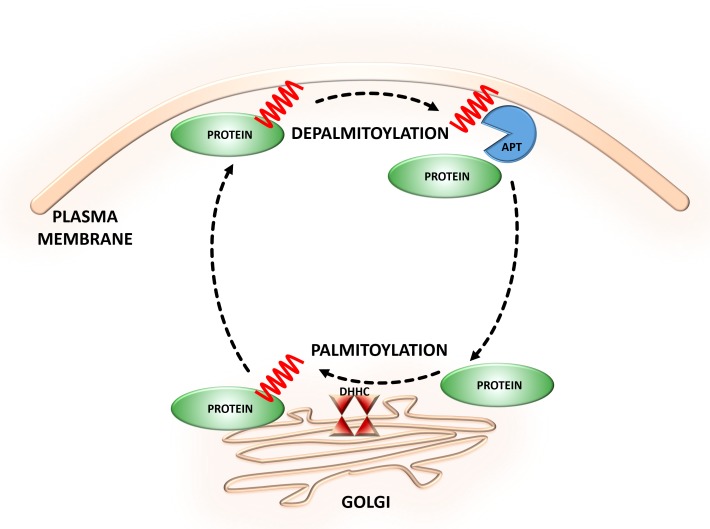
Dynamic *S*-palmitoylation of proteins. *S*-Palmitoylation is a reversible and dynamic protein modification that is mediated by DHHC-palmitoyltransferases and acyl protein thioesterases that attach to and remove palmitate from cysteine residues of proteins, respectively.

Protein *S*-acylation is a lipid modification that involves the covalent attachment of long-chain fatty acids to thiol groups of cysteine (Cys) residues through thioester linkage. This type of protein modification is commonly referred to as *S*-palmitoylation (S-PALM) because Cys residues are predominately acylated with palmitic acid (16-C), although Cys may also be modified with both longer-chain fatty acids and unsaturated fatty acids (Xu, 2011; Verpelli et al., 2012). Cys *S*-fatty-acylation is a reversible posttranslational modification (PTM) that can dynamically regulate protein stability, trafficking, and activity (Ardiles et al., 2012; Yokoi et al., 2012). S-PALM is mediated by the aspartate-histidine-histidine-cysteine (DHHC) family of protein acyltransferases (PATs) and different classes of thioesterases (Kang et al., 2008; Conibear and Davis, 2010; Fukata and Fukata, 2010; Greaves and Chamberlain, 2011; Fukata et al., 2013) (**Figure 1**). The mechanisms that underlie site-selective S-PALM and the potential role of specific sequence motifs remain largely unknown. The large substrate diversity is related to the large array of PATs (Noritake et al., 2009). Twenty-three distinct DHHC among PATs have been identified in mammalian cells, which mediate the bulk of protein palmitoylation (Noritake et al., 2009; Ohno et al., 2012). In contrast, the existence of depalmitoylating enzymes remains controversial. To date, seven proteins—ABHD17, ABHD12, ABHD13, Lypla1, Lypla2, Apt1, and Ppt1—have been identified as candidates for several limited substrates, such as H-Ras, PSD-95, and Gα (Lin and Conibear, 2015; Yokoi et al., 2016; Won et al., 2018). However, remaining unknown is whether these enzymes are able to depalmitoylate other *S*-palmitoylated substrates.

The unique reversible nature of this PTM allows palmitoylated proteins for dynamic relocation between different cellular compartments (Fukata et al., 2013; Holland and Thomas, 2017; Tortosa and Hoogenraad, 2018). Owing to the palmitate hydrophobicity, modified proteins normally associate with the membranes of various organelles and facilitate trafficking between these organelles (Kandasamy and Larson, 2006; Greaves et al., 2009; Aicart-Ramos et al., 2011). Features of the palmitoylation domains that are important for membrane targeting include the spacing of palmitoylated cysteines and the presence of basic amino acids downstream of the cysteines (Salaun et al., 2010). However, it is still unclear how these specific features of *S*-palmitoylation contribute to protein sorting.

The emerging picture is that palmitoylation may be crucial for directing proteins to distinct transport vesicles and allows a selection of specific cytosolic proteins to bind on secretory vesicles (Tortosa and Hoogenraad, 2018). Secretory vesicles have been found in both axons and dendrites of neurons and distribute modified proteins to all neuronal compartments (Lasiecka and Winckler, 2011). Proteins are packaged into distinct transport carriers and transported to the specific axonal or dendritic compartments (de Wit et al., 2006). There, sorting signals, within the cytoplasmic region of transmembrane proteins, are recognized by sorting adaptors and next concentrate them in the appropriate vesicles (Chum et al., 2016; Tortosa and Hoogenraad, 2018). Various sorting motifs, such as tyrosine- or dileucine-based motifs, are present in the majority of proteins, and interact with different adaptor protein complexes (Bonifacino and Dell’Angelica, 1999; Vergarajauregui and Puertollano, 2006; Pandey, 2009). Interestingly, different substrate proteins can co-traffic on a single vesicle (Tortosa et al., 2017). The secretory vesicles which transport palmitoylated SCG10 and N-Ras proteins also contain other palmitoylated proteins such as the microtubule associated protein MAP6 (Tortosa et al., 2017).

It is still unclear whether palmitoylated proteins influence secretory vesicle fusion. On the other hand, the selective retention of proteins at the final destination depends on the local activity of depalmitoylating enzymes. In neurons, palmitoylated MAP6 is bound to secretory vesicles and transported to all neurites. However, neuronal polarization leads to MAP6 depalmitoylation, resulting in membrane release of MAP6 from vesicles and its accumulation in the newly formed axon (Tortosa et al., 2017).

Numerous studies have shown that neuronal proteins are modified by palmitoylation. Among these, we can distinguish scaffolding proteins, ion channels, signaling proteins, enzymes, cell adhesion molecules, and many G-protein-coupled receptors (Kang et al., 2008; Prescott et al., 2009; Salaun et al., 2010). Moreover, S-PALM is thought to be a particularly important regulator of synaptic plasticity, a biological process by which diverse neuronal activity modulates protein composition at the synapse, leading to changes in synaptic strength (Washbourne, 2004; Kang et al., 2008). Several S-PALM target synaptic proteins contribute to the dynamic and functional modulation of synapses (Washbourne, 2004; Kang et al., 2008; Noritake et al., 2009; Fukata and Fukata, 2010; Yokoi et al., 2012; Fukata et al., 2013). The purpose of this review is to demonstrate that the S-PALM-based regulation of synaptic proteins is not accidental, but highly defined to specific molecules and pathways that can be modified in neurological disorders. Thus we chose the major synaptic proteins with different molecular functions such as scaffolding proteins, receptors, kinases, and signaling molecules modulated by S-PALM. We also provide examples of neurological disorders in which the role of S-PALM has been confirmed. We then discuss the methods that are available for detecting and analyzing S-PALM, together with their advantages and disadvantages.

## Palmitoylated Synaptic Proteins

### PSD-95

**FIGURE 2:**
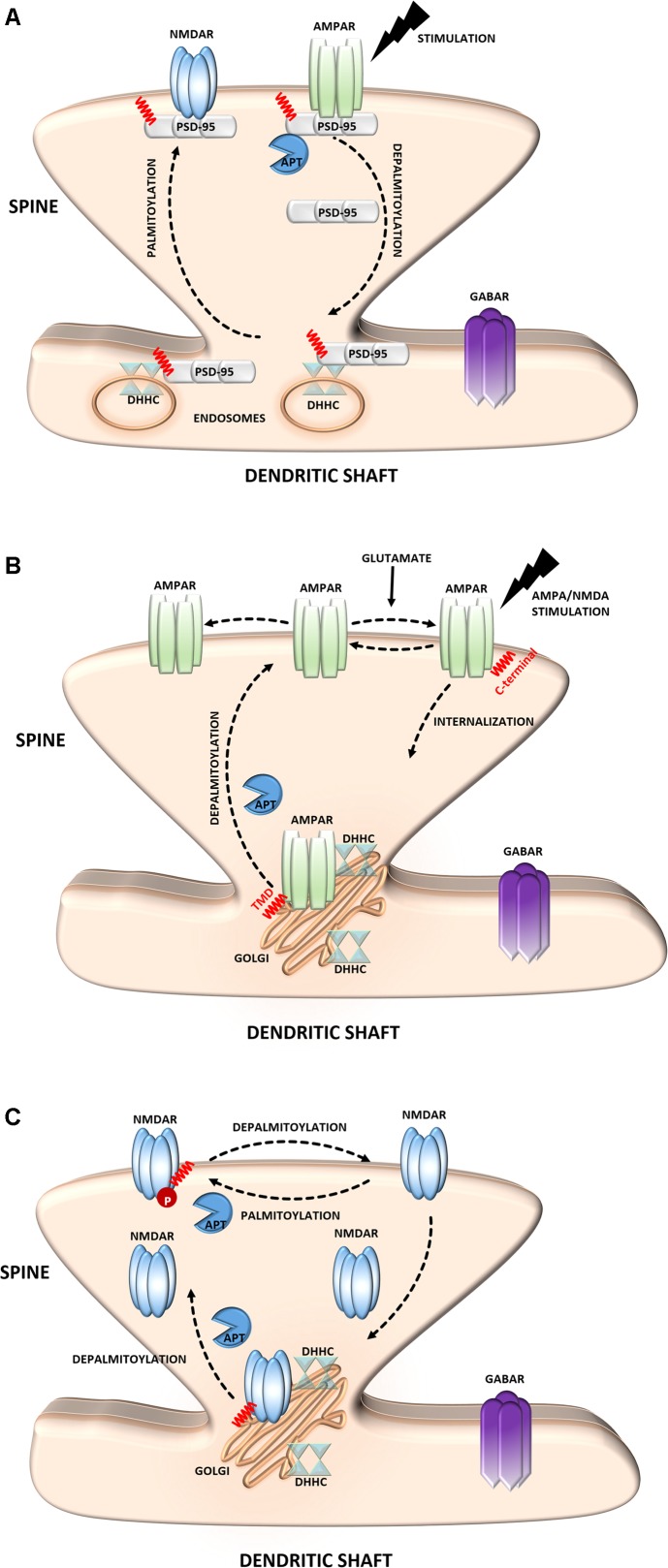
Schematic overview of *S*-palmitoylated proteins that are crucial for excitatory synapse function. **(A)** PSD-95. Dendritically localized DHHC mediates activity-sensitive palmitoylation of PSD-95 and its synaptic clustering. Palmitoylated PSD-95 regulates glutamate receptors (NMDAR and AMPAR) recruitment at postsynaptic sites, thus affecting synaptic efficacy. Enhanced stimulation of glutamate receptors leads to depalmitoylation of PSD-95 and its dissociation from the PSD. As a result of PSD-95 depalmitoylation, the glutamate receptors internalize and the synapse strength decreases. **(B)** AMPAR. Palmitoylation of GluA1/A2 subunits on the *transmembrane domain (TMD) accumulates AMPAR in the Golgi apparatus, whereas depalmitoylation triggers receptor trafficking to the cell surface. C-terminal palmitoylation of GluA1/A2 does not influence the glutamate-dependent steady-state surface expression of AMPAR. On the other hand, palmitoylated form of the receptor is more sensitive to AMPA- and NMDA-induced receptor internalization. **(C)** NMDAR. Palmitoylation of the NMDARs on a membrane-proximal region of the NR2A/2B subunits ensures proper surface delivery and increases the stability of NMDAR on the postsynaptic membrane via tyrosine phosphorylation. In contrast, palmitoylation of the NR2A/2B subunits in the middle of C-terminal region increases accumulation of the NMDAR in the Golgi apparatus and decreases receptor surface expression. In turn, depalmitoylation of the NR2A/2B subunits regulates release of NMDA receptors from the Golgi for surface delivery.*

As the most abundant scaffolding protein in the postsynaptic density (PSD) at excitatory synapses, PSD-95 is thought to be responsible for targeting and stabilizing various proteins, including glutamate receptors [*N*-methyl-D-aspartate receptors (NMDARs) and α-amino-3-hydroxy-5-methyl-4-isoxazolepropionic acid (AMPA)], neuroligin, potassium channels, and other scaffolding proteins, to the postsynaptic membrane (Xu, 2011; Verpelli et al., 2012). Therefore, PSD-95 significantly contributes to neurotransmission and synaptic strength. PSD-95 is palmitoylated at two cysteine residues, Cys3 and Cys5 (Hsueh and Sheng, 1999; Noritake et al., 2009; Ho et al., 2011). The palmitoylation of this protein is essential for its clustering at the PSD and subsequent membrane scaffolding function (Jeyifous et al., 2016) (**Figure 2A**). Notably, the palmitate turnover on PSD-95 is regulated by neuronal activity (el-Husseini and Bredt, 2002; Yokoi et al., 2012). The greater stimulation of glutamate receptors results in the depalmitoylation of PSD-95 and its dissociation from the PSD (Thomas and Huganir, 2013). This leads to the internalization of glutamate receptors and downregulation of synaptic strength. Lower neuronal activity induces the palmitoylation of PSD-95 and promotes the trafficking of PSD-95 and glutamate receptors to the postsynaptic membrane (Washbourne, 2004; Ho et al., 2011; Thomas and Huganir, 2013; Jeyifous et al., 2016). PSD-95 has shown apparent palmitate cycling. This process is distinctively regulated by combination of palmitoylating and depalmitoylating enzymes in response to extracellular stimuli in neuros (Yokoi et al., 2016). Thus, the glutamate-induced palmitoylation cycle of PSD-95 is of key importance to synaptic strength and plasticity.

### Glutamate Receptors

Based on their pharmacological properties ionotropic glutamate receptors are divided into three classes of: α-amino-3-hydroxyl-5-methyl-4-isoxazole-propionate receptors (AMPARs), NMDARs, and kainate receptors (KARs).

α-Amino-3-hydroxyl-5-methyl-4-isoxazole-propionate receptors mediate the majority of fast excitatory synaptic transmission in the mammalian brain (Sheng and Kim, 2011). The dynamic regulation of AMPARs at the synapse depends on interactions with PSD-95 and self-palmitoylation. AMPARs are ligand-gated ion channels that are composed of combinations of four separate subunits (GluA1-4) (Henley and Wilkinson, 2013). Notably, the S-PALM of these receptors is a subunit-specific process that affects their localization and trafficking (Hayashi et al., 2005; Kang et al., 2008; Lin et al., 2009; Yang et al., 2009) (**Figure 2B**). Synaptic AMPARs predominantly comprise combinations of GluA1 and GluA2, which are directly regulated by S-PALM. Two palmitoylation sites of these subunits have been described. One site, cysteine 610, in the second transmembrane domain, is similar for both subunits, whereas the other site, on their C-terminal regions is different. GluA1 and GluA2 are palmitoylated at cysteine 811 and cysteine 836, respectively (Hayashi et al., 2005; Yang et al., 2009; Henley and Wilkinson, 2013). Palmitoylation in the transmembrane domain causes accumulation of the receptor in the Golgi apparatus and inhibits its surface expression. On the other hand, palmitoylation of the C-terminal site does not affect the steady-state plasma membrane expression of the AMPAR. Interestingly, neuronal activity highly regulates the palmitoylation of GluA1 and GluA2. The blockade of neuronal activity increases the amount of palmitoylated GluA2 but not GluA1 in the postsynaptic membrane, whereas strong excitatory stimulation accelerates depalmitoylation but in a subunit-specific manner (Hayashi et al., 2005; Henley and Wilkinson, 2013; Han et al., 2015).

*N*-methyl-D-aspartate receptors are another type of glutamate receptor and ion channel that are found at most excitatory synapses (Sanhueza et al., 2011; Xu, 2011). NMDARs consist of three families of homologous subunits: NR1, NR2, and NR3. The NMDAR NR2A and NR2B subunits are palmitoylated in their C-terminal region (Hayashi et al., 2009; Mattison et al., 2012) (**Figure 2C**). Palmitoylation on a membrane-proximal region of NMDARs increases tyrosine phosphorylation, leading to enhanced stable surface expression of the receptor in neurons. In contrast, palmitoylation in the middle of the C-terminal region leads to the accumulation of NMDARs in the Golgi apparatus and decreases receptor surface expression (Hayashi et al., 2009). Similar to AMPARs, NMDAR palmitoylation is specific to a particular subunit and dynamically regulated in an activity-dependent manner. This PTM differentially modulates NMDAR localization and might be crucial for proper neuronal function.

Kainate receptors are engaged in the regulation of both excitatory and inhibitory neurotransmission. Similarly to AMPARs and NMDARs, KARs trafficking and plasma membrane expression are modulated by interactions with intracellular proteins. Five types of KAR subunits, GluK1, GluK2, GluK3, GluK4, and GluK5 can be arranged in different ways to form a tetramer, a four subunit receptor (Lerma, 2006). GluK2 is palmitoylated at two C-terminal tail cysteine residues Cys858 and Cys871 (Copits and Swanson, 2013). In contrast to AMPARs, palmitoylation of GluK2 promotes association of KARs with protein 4.1N and stabilizes neuronal surface expression of KARs due to reduced constitutive internalization of these receptors. Moreover, palmitoylation of Cys871 inhibits phosphorylation of GluK2 by protein kinase C (PKC) and thereby modulates KAR function.

### Dopamine Receptors

Dopamine is acting as a modulator of variety of neuronal functions, such as cognition, emotion, and motor activity (Wise, 2004; Zhang et al., 2016; Volkow et al., 2017). It activates five dopamine receptors, D_1_ through D_5_, which are classified into two subtypes, D_1_-like (D_1_ and D_5_) and D_2_-like (D_2_, D_3_, and D_4_) dopamine receptors. Activation of D_1_-like receptors may lead to excitation or inhibition of the target neuron while the activation of D_2_-like receptors usually is linked with neuronal inhibition.

Among D_1_-like receptor subtypes only *S*-palmitoylation of D_1_ receptor (D_1_R) was experimentally identified (Jin et al., 1999). Palmitoylation may target D_1_R through selective endocytic pathways (clathrin or caveolar) (Kong et al., 2011). It was demonstrated that a depalmitoylated D_1_R is internalized faster than palmitoylated one. Thus, palmitoylation may be involved in directing the D_1_R to the slower caveolae-dependent endocytic route (Kong et al., 2011). Instead, the functional significance of the homologous cysteines in D_5_R has to be elucidated.

All D_2_-like dopamine receptors contain C-terminal conserved cysteines which serve as the sites of palmitoylation. Some *in vitro* studies with insect cells (Sf9) and mammalian cells (HEK293) have shown that the short (D_2S_) and long (D_2L_) isoforms of D_2_R are palmitoylated (Ng et al., 1994; Grünewald et al., 1996; Ebersole et al., 2015). Moreover, it was demonstrated that this modification affects the plasma membrane expression and stability of D_2L_. However, the opposite results were also reported showing that the short isoform of D_2_R (D_2S_) is not palmitoylated and that the inhibition of palmitoylation does not affect receptor functions, including surface expression (Zhang et al., 2016). Other study has shown that D_3_R receptor is also *S*-palmitoylated. D_3_R palmitoylation was found to be required for the proper expression of this receptor on the cell surface, endocytosis, agonist binding, and receptor tolerance. Also third member of D_2_-like subtype of dopamine receptors, D_4_R, has been identified in *S*-palmitoylated form. Similarly to D_3_R, *S*-palmitoylation of D_4_R is required for its proper localization within the plasma membrane, endocytosis, and signaling (Zhang and Kim, 2016).

### GABA Receptors

**FIGURE 3:**
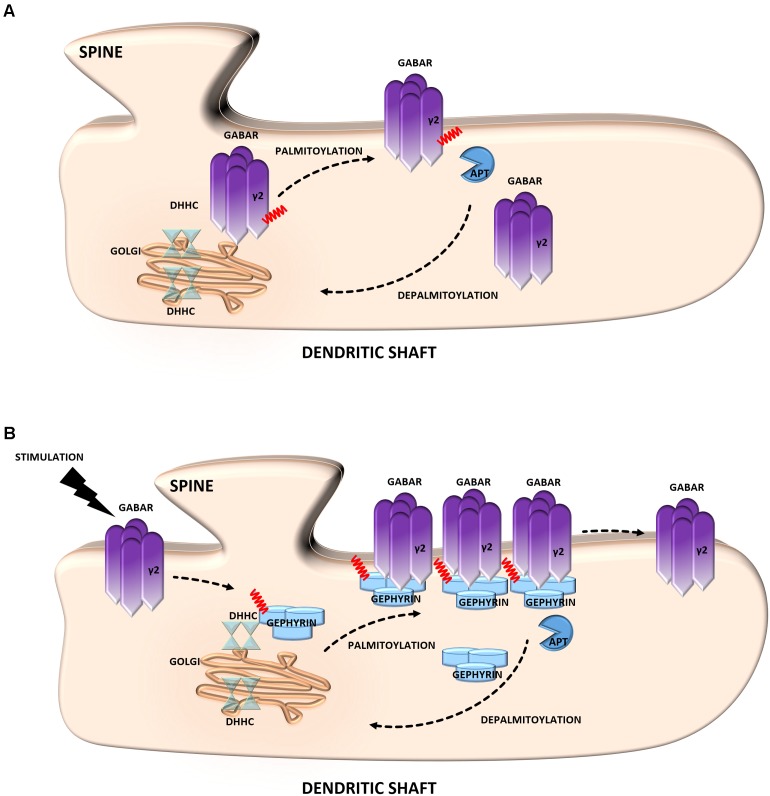
Schematic overview of *S*-palmitoylated proteins that are crucial for inhibitory synapse function. **(A)** GABAR. Palmitoylation of the γ2 subunit determines the stability and mobility of receptors in the postsynaptic membrane and is required for normal GABAergic inhibitory transmission. **(B)** Gephyrin. Gephyrin, palmitoylated on two cysteine residues forms stable clusters at the postsynaptic membrane. Thereby increasing the strength of GABAergic transmission. Stimulation of GABAergic transmission leads to gephyrin palmitoylation and membrane association, ultimately increasing the size of gephyrin clusters.

A striking example of the neuronal palmitoyl regulation of inhibitory synapses is provided by γ-aminobutyric acid type A receptors (GABA_A_Rs) (Keller et al., 2004; Fang et al., 2006; Luscher et al., 2011). These receptors are heteropentameric GABA-gated chloride channels that consist of subunits from seven homologous subclasses. The majority of these receptors contain α1–6, β1–3, and γ1–3 subunits. Of particular interest is the γ2 subunit, which is required for targeting GABA_A_Rs to inhibitory synapses (Luscher et al., 2011). Notably, this subunit is *S*-palmitoylated on multiple cysteine residues in the cytoplasmic region, and this mechanism regulates the normal expression of GABA_A_Rs at the cell surface of neurons (Vithlani et al., 2011) (**Figure 3A**). Using the SOS recruitment-yeast two-hybrid system it was shown that the γ2 subunit of GABA_A_R is a substrate for the palmitoyltransferase, Golgi-specific DHHC zinc finger domain protein (GODZ) (Keller et al., 2004). Disrupting GODZ function or expression levels using dominant negative or RNAi approaches resulted in a significant reduction in the amplitude of mIPSCs attributed to a decrease in postsynaptic GABA_A_Rs number. Likewise, immunofluorescent analyses of neurons transfected with a GODZ-specific shRNA vectors revealed a marked reduction of punctate γ2 subunit immunoreactivity as well as loss of postsynaptic gephyrin staining compared with neurons transfected with control shRNA (Fang et al., 2006). From these studies it is evident that the S-PALM of GABA_A_Rs is implicated in the dynamic regulation of membrane localization and function of these receptors, thereby modulating GABAergic inhibitory transmission.

### Gephyrin

Gephyrin is an inhibitory synapse scaffolding protein that anchors glycine and major subsets of GABA_A_Rs in the postsynaptic membrane (Choii and Ko, 2015). It was found to be palmitoylated at two cysteine residues (Cys212 and Cys284) that are critical for both the association and stable clustering of gephyrin at GABAergic synapses (Dejanovic et al., 2014) (**Figure 3B**). Furthermore, gephyrin S-PALM is regulated by GABA_A_R activity. The activation of these receptors by agonists increases the palmitoylation of gephyrin and its membrane affinity, whereas the blockade of GABAergic transmission evokes opposite effects (Battaglia et al., 2018).

### LIM Kinase-1

**FIGURE 4:**
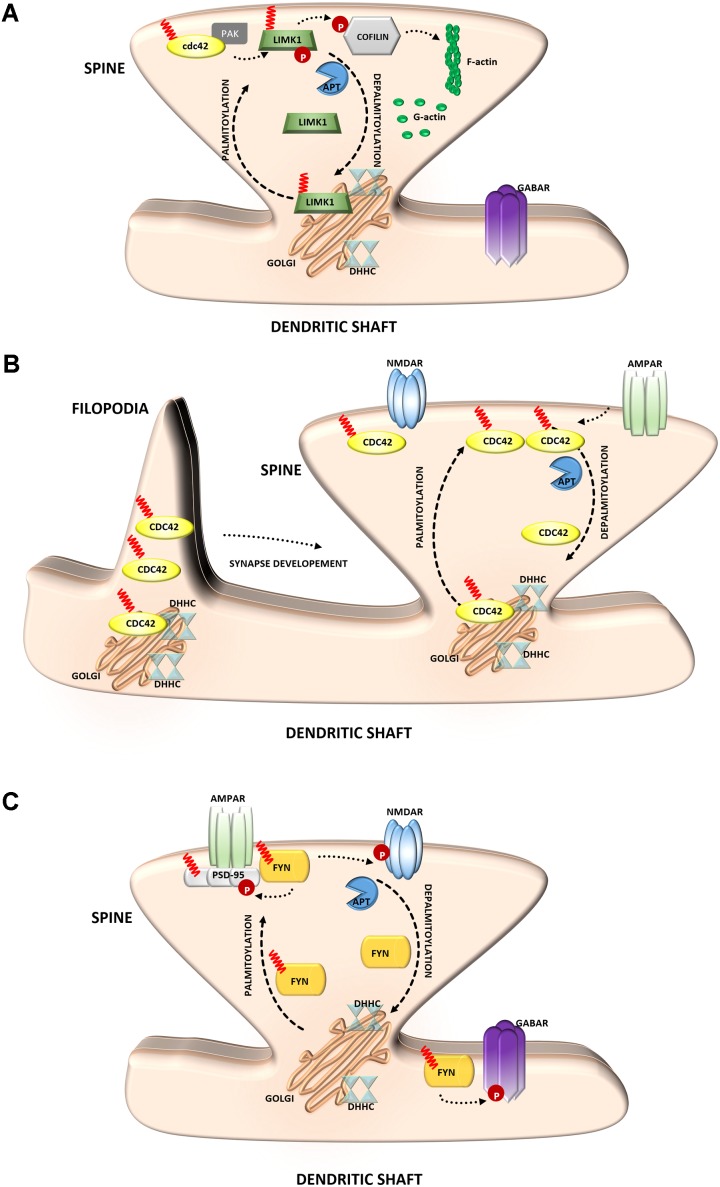
Schematic overview of *S*-palmitoylated signaling molecules that are important for synapse function. **(A)** LIM kinase-1. Palmitoylation targets LIMK1 to the spine membrane, where it is phosphorylated by membrane-bound activators such as Cdc42/PAK. Palmitoylated LIMK1 phosphorylates cofilin and thus regulates spine-specific actin polymerization and morphological plasticity. In contrast, non-palmitoylated LIMK1 remains inactive **(B)** GTPase Cdc42. Palmitoylated Cdc42 accumulates in dendritic filopodia modulating their maturation and is strongly concentrated in dendritic spines. In response to enhanced neuronal activity Cdc42 is depalmitoylated and dissociated from the spine. **(C)** Tyrosine-protein Fyn kinase. Palmitoylation of Fyn is required for its membrane localization and thus proper action. Palmitoylated Fyn phosphorylates various substrates important for the accurate synaptic functioning, e.g., PSD-95, NMDAR, GABAR.

The important actin regulatory protein LIM kinase-1 (LIMK1) phosphorylates and inactivates the actin-severing protein cofilin, thus promoting actin polymerization (Yang et al., 1998). LIMK1 appears to be particularly important for actin regulation in dendritic spines on which most excitatory synapses are formed (Hotulainen and Hoogenraad, 2010). Changes in the size and shape of individual spines are closely associated with synaptic efficiency (Hotulainen and Hoogenraad, 2010; Bosch and Hayashi, 2012; Bosch et al., 2014). Dynamic modulation of the actin cytoskeleton enables rapid changes in the shape or volume of dendritic spines in response to stimulation (Honkura et al., 2008). This process requires the precise spatial regulation of proteins that increase actin polymerization. This mechanism was shown to consist of the palmitoylation of LIMK1 (**Figure 4A**). LIMK1 is palmitoylated at adjacent cysteine residues, Cys7 and Cys8, and this PTM is crucial for LIMK1 function because it not only controls the targeting of LIMK1 to spines but is also essential for the activation of LIMK1 by its membrane-localized upstream activator PAK (George et al., 2015). These results show that palmitoyl-LIMK1 is critical for normal spine actin turnover, activity-dependent structural plasticity, and long-term spine stability.

### Cdc42

The Rho family guanosine triphosphatase (GTPase) Cdc42 regulates cytoskeletal organization and membrane trafficking in such physiological processes as cell proliferation, motility, and polarity. Interestingly, Cdc42 is rapidly and locally activated in dendritic spine heads by potentiating stimuli, reflecting its unique contribution to the regulation of spine morphology (Woolfrey and Srivastava, 2016). Cdc42 is expressed in the two splice variants: canonical (which is prenylated) and a brain-specific form (which can be palmitoylated). These two isoforms have distinct dendritic localizations. Cdc42-prenyl is distributed throughout the dendrite, at both the dendritic spine and shaft. Cdc42-palm is more heavily localized to dendritic spines. The Cdc42-palm isoform was shown to be required for the extension of dendritic filopodia, the key stage of spine formation and synaptogenesis (Kang et al., 2008) (**Figure 4B**). Interestingly, glutamate induces the depalmitoylation and rapid dislocation of Cdc42 from dendritic spines. Thus, the concentration of Cdc42 in spines can be rapidly modified by neuronal activity in a palmitoylation-dependent manner, which may provide dynamic changes in spine morphology (Wirth et al., 2013; Moutin et al., 2017).

### Tyrosine-Protein Fyn Kinase

Several studies have indicated a role for palmitoylation in signaling by Src-family kinases (Sato et al., 2009) (**Figure 4C**). The influence of Fyn kinase on neuronal signaling has long been known. The importance of palmitoylation for Fyn membrane targeting suggests that neuronal Fyn function requires its palmitoylation-dependent synaptic membrane association (Gottlieb-Abraham et al., 2016). Notably, neuronal Fyn substrates include several transmembrane or membrane-associated proteins that are key components of excitatory synapses. One of the best-known neuronal Fyn substrates is the NR2B subunit of the NMDA-type glutamate receptor, and phosphorylation by Fyn has been proposed to stabilize this subunit at the neuronal cell surface (Trepanier et al., 2012). The synaptic scaffolding proteins PSD-95 and PSD-93 are also Fyn binding partners and substrates (Nada et al., 2003). Interestingly, the important role of Fyn in signaling is not limited to excitatory synapses because Fyn also likely phosphorylates GABA receptors that are localized to inhibitory synapses (Jurd et al., 2010).

## Palmitoylation and Neurological Diseases

**FIGURE 5:**
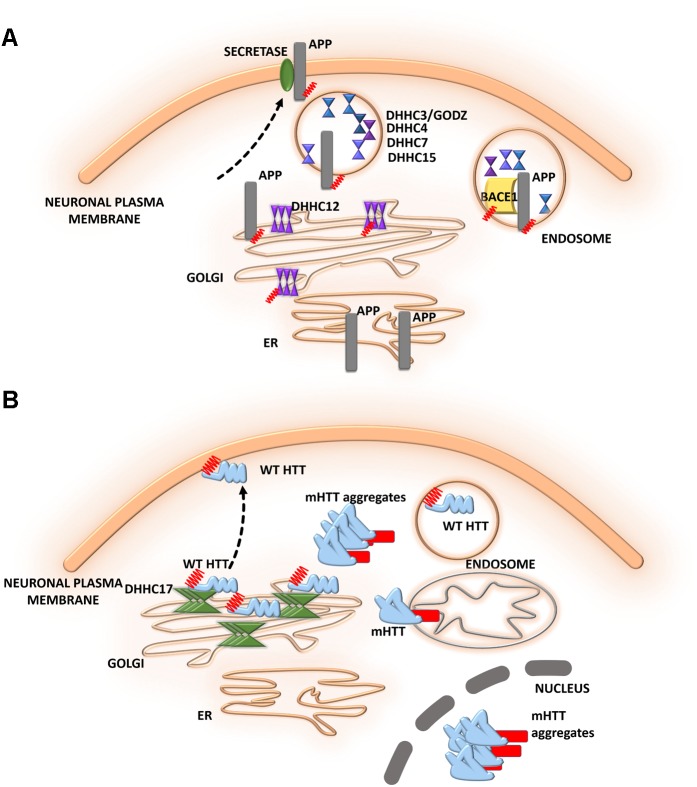
Schematic overview of *S*-palmitoylated proteins and DHHC enzymes related to neurodegenerative diseases. **(A)** Alzheimer’s disease. S-PALM APP is enriched in lipid rafts where it is cleaved by BACE1. DHHC3/GODZ, DHHC4, DHHC7, and DHHC15 promote the S-PALM of BACE1, which may facilitate the amyloidogenic process. Additionally, DHHC12 is involved in the regulation of APP localization, trafficking, and metabolism. **(B)** Huntington’s disease. The S-PALM of HTT is crucial for protein localization and trafficking. PolyQ expansion in HTT leads to lower affinity of the protein for its specific DHHC17, resulting in a reduction of HTT palmitoylation. Ion-resistant mutant HTT exhibited an increase in toxicity and a propensity to form aggregates.

**FIGURE 6:**
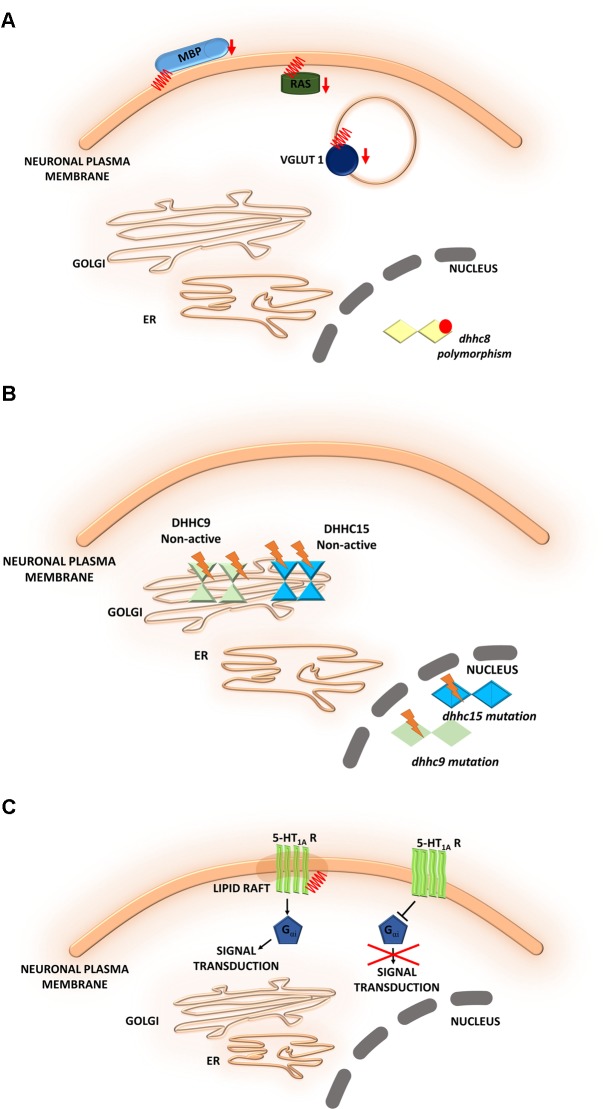
Schematic overview of *S*-palmitoylated proteins and DHHC enzymes related to neuropsychiatric diseases. **(A)** Schizophrenia. A reduction of the palmitoylation of many proteins, such as vesicular glutamate transporter 1 (VGLUT1), the small GTPase Ras, and myelin basic protein (MBP), has been observed in the dorsolateral prefrontal cortex of schizophrenia patients. DHHC8 is located in the microdeletion region of chromosome 22q11 and may increase the risk of schizophrenia. **(B)** Intellectual disability. DHHC15 and DHHC9 are implicated in X chromosome-linked ID (XLID). Genetic mutations of chromosome X led to the disruption of transcription of the *dhhc15* gene in a female patient with severe non-syndromic XLID. Four mutations of the *dhhc9* gene were identified in patients with XLID. **(C)** Major depressive disorder. The S-PALM of 5-HT receptors may be directly involved in receptor isomerization from an inactive to an active form and may represent a general feature that regulates constitutive receptor activity. S-PALM is a targeting signal that is responsible for the retention of 5-HT_1A_ receptors in lipid rafts, which represent scaffold platforms for receptor-mediated signaling.

Numerous studies have demonstrated that defects in protein palmitoylation and the aberrant activity of palmitoylating and depalmitoylating enzymes are associated with a wide range of human neurological diseases. In many cases, pathophysiology correlates with changes in the pattern of this modification at the specific protein target level of specific modified sites (**Figures 5, 6**).

### Alzheimer’s Disease

Alzheimer’s disease (AD) is one of the most common neurodegenerative disorders. The neurodegenerative process in AD is characterized by synaptic damage, accompanied by neuronal loss (Ardiles et al., 2012). Several lines of evidence support the notion that synaptic pathology and defective neurogenesis in AD are related to the progressive abnormal accumulation of amyloid β (Aβ) oligomers, together with the hyperphosphorylation of microtubule-associated protein tau (Walsh and Selkoe, 2007). These toxic Aβ oligomers are derived from the sequential proteolytic processing of amyloid precursor protein (APP). This process involves several enzymes, including BACE1 (β-site APP cleaving enzyme) and β- and γ-secretases. Bhattacharyya et al. (2013, 2016) reported that two N-terminal cysteine residues of APP (Cys186 and Cys187) undergo S-PALM. S-PALM APP is enriched in membrane lipid rafts and forms dimers, leading to elevations of BACE1-mediated cleavage of the protein (Bhattacharyya et al., 2013). These results indicate that APP palmitoylation enhances Aβ peptide production through the amyloidogenic pathway. A radiolabeling method, combined with point mutations of cysteines, showed that BACE1 is palmitoylated at four cysteine residues (Cys474, Cys478, Cys482, and Cys485). Although palmitoylated BACE1 is preferentially targeted to lipid rafts, this is not directly related to APP cleavage (Motoki et al., 2012). Studies that used knock-in mice (with cysteine-to-alanine substitution at the palmitoylated sites) that selectively lacked BACE1 S-PALM reported that the palmitoylation-deficient mutant of BACE1 exhibited non-raft localization but no changes in the BACE1-dependent APP processing or secretion of Aβ (Andrew et al., 2017). To date, the contribution of BACE1 S-PALM to Aβ production and AD progression is still unclear and requires further investigation.

The dysregulation of enzymes that control protein palmitoylation may also lead to the development of AD. DHHC12 has been shown to be involved in regulating APP localization, trafficking, and metabolism (Cho and Park, 2016). Enzymatic activity inhibits APP metabolism, and the production of Aβ leads to retaining the APP protein in the Golgi apparatus, thus preventing trafficking to the trans-Golgi network and membrane. Five different DHHC enzymes–DHHC3/GODZ, DHHC4, DHHC7, DHHC15, and DHHC20–were shown to promote the palmitoylation of BACE1, which may facilitate the amyloidogenic processing of APP by targeting BACE1 to lipid rafts (Levy et al., 2011). This protective function of DHHC12 was hypothesized to be exerted by the palmitoylation of its substrates other than APP (**Figure 5A**).

### Huntington’s Disease

The role of S-PALM in Huntington’s disease (HD) is less well defined, but some research has shed light on the ways in which reversible palmitoylation may contribute to the pathogenesis of HD (**Figure 5B**). Huntington’s disease is a neurodegenerative disease that is characterized by cognitive decline, motor dysfunction, and psychiatric disturbances (Young et al., 2012; Wan et al., 2013; Sanders and Hayden, 2015). Huntington’s disease is caused by a mutation of the *huntingtin* gene, resulting in an abnormal huntingtin protein (HTT) with a long polyglutamine, poly(Q) tail in the N-terminal region (Sharma and Priya, 2017). At the histopathological level, the loss of medium spiny neurons and smaller striatal volume are observed. Huntingtin protein was shown to be highly expressed in neurons. Using the PULSE-CHASE radiolabeling method, a previous study found that HTT is palmitoylated at Cys214 under physiological conditions, which is crucial for protein localization and trafficking (Sanders and Hayden, 2015). PolyQ expansion in HTT was also shown to lead to lower affinity of the protein for its specific DHHC17, resulting in a reduction of HTT palmitoylation (Butland et al., 2014). Although palmitoylation-resistant mutant HTT exhibited an increase in toxicity and a propensity to form aggregates, palmitoylation may affect the pathogenesis of HD.

### Schizophrenia

Schizophrenia is a chronic, severe psychiatric disorder that is often characterized by abnormal social behavior and prevalent cognitive deficits. Microdeletions in chromosome 22q11 have been described as a main factor that contributes to the development of neuropsychiatric symptoms of schizophrenia. The gene that encodes a palmitoylating enzyme, DHHC8, is located in the microdeletion region of chromosome 22q11 and may increase the risk of schizophrenia (Xu et al., 2008). Polymorphisms of the *dhhc8* gene have been reported to be associated with the risk of schizophrenia, but these associations appeared to depend on ethnicity (Liu et al., 2002; Faul et al., 2005). Therefore, the relationship between *dhhc8* gene polymorphisms and schizophrenia remains controversial (Mukai et al., 2008, 2015). Interestingly, other proteomic studies have reported a reduction of the palmitoylation of many proteins, such as vesicular glutamate transporter 1 (VGLUT1), the small GTPase Ras, and myelin basic protein (MBP), in the dorsolateral prefrontal cortex in schizophrenia patients (Pinner et al., 2016) (**Figure 6A**).

### Intellectual Disability

Intellectual disability is another neurodevelopmental disorder that is characterized by an early onset (<18 years of age). S-PALM has been implicated in intellectual disability. Intellectual disability can be described as limitations in cognitive and adaptive function, such as daily living and social and communication skills. It was shown that palmitoylating enzymes, namely DHHC15 and DHHC9, are implicated in X chromosome-linked intellectual disability (XLID) (Raymond et al., 2007; Tarpey et al., 2009; Hu et al., 2016). Genetic mutations of chromosome X were shown to lead to the disruption of transcription of the *dhhc15* gene in a female patient with severe non-syndromic XLID (Raymond et al., 2007; Hu et al., 2016). Additionally, four mutations of the *dhhc9* gene were identified in four patients with XLID (Raymond et al., 2007). These mutations affected the level of DHHC9 autopalmitoylation, thereby modulating the palmitoylation of its target proteins that may be involved in intellectual development (Mitchell et al., 2014) (**Figure 6B**). Therefore, it is extremely important to identify novel target proteins of DHHC9 that are associated with intellectual disability to facilitate molecular diagnosis and therapeutic treatment (Masurel-Paulet et al., 2014).

### Major Depressive Disorder

Major depressive disorder (MDD) is a common psychiatric disorder that comprises a spectrum of symptoms, such as deficits in cognitive, psychomotor, and emotional processes (Rigucci et al., 2010; Bennett, 2011). One class of *S*-palmitoylated proteins, serotonin [5-hydroxytryptamine (5-HT)] receptors, has been linked to MDD. Palmitoylation was experimentally confirmed for following 5-HT receptors: 5-HT_1A_, 5-HT_1B_, 5-HT_3A_, 5-HT_4_, and 5-HT_7_ (Papoucheva et al., 2004; Ponimaskin et al., 2005; Renner et al., 2007; Kobe et al., 2008; Kvachnina et al., 2009; Gorinski and Ponimaskin, 2013).

5-HT_1A_ receptor is modified by covalently attached palmitate and that palmitoylation is irreversible and insensitive to agonist stimulation (Papoucheva et al., 2004). Obtained data suggest that the S-PALM of 5-HT_1A_ receptors may be directly involved in receptor isomerization from an inactive form to an active form and may represent a general feature that regulates constitutive receptor activity (Renner et al., 2007; Gorinski and Ponimaskin, 2013) (**Figure 6C**). Furthermore 5-HT_1A_ receptor palmitoylation serves as a targeting signal responsible for localization of this receptor in membrane rafts. More importantly, the 5-HT_1A_ raft localization seems to be involved in receptor-mediated signaling. It was shown that palmitoylation of 5-HT_1A_ receptor is necessary for G_αi_-protein coupling and effector signaling where communication between receptors and G_αi_-subunits was completely abolished after blocking of this modification (Papoucheva et al., 2004). Additionally, non-palmitoylated mutants of 5-HT_1A_ couldn’t inhibit cAMP formation, indicating that palmitoylation of the 5-HT_1A_ receptor is critical for the enabling of Gi-protein coupling/effector signaling. Dysregulation of the serotonergic system has been observed in the brains of MDD patients and rodent models of depression and anxiety (Hajszan et al., 2009). The effect of 5-HT receptor S-PALM on structural plasticity that underlies depression remains to be elucidated.

## Methods for Detecting S-Palm

Despite accumulating evidence of the key role of S-PALM in physiological neuronal regulation and neuropathological processes, technical difficulties have hindered the detection and analysis of S-PALM. Such difficulties are further exacerbated by the lack of identification of an S-PALM consensus motif for prediction. The range of involvement of this PTM in synaptic plasticity appears to be underestimated. Therefore, determining the precise levels and sites of protein S-PALM is crucial for understanding the ways in which this dynamic lipid modification is regulated during neuronal activity.

Below we critically review the methods that are currently used to detect and enrich protein S-PALM. These methods can generally be divided into two major groups. The first group includes metabolic labeling methods that exploit synthetic analogs of fatty acids with biorthogonal handles (so-called chemical reporters) that are incorporated into proteins to Cys residues. The second group contains Acyl Biotin Exchange (ABE), Acyl Resin-Assisted Capture (Acyl-RAC), and Acyl-PEG Exchange (APE). All of these methods are based on selective thioester liberation and *S*-acylated residue labeling and thus have the potential to capture the full *S*-acyl proteome but not a specific fatty acid, such as palmitate. The methods that are used as chemical reporters can be applied to label proteins with fast fatty-acid turnover (Conibear and Davis, 2010).

## Metabolic Labeling

### Metabolic Radioactive Labeling

This is one of the most commonly used S-PALM detection methods. The oldest approach was radioactive labeling, first introduced by Schmidt et al. (1979). In the standard experiment, cells are transfected with a plasmid or viral vectors for specific protein overexpression and subsequently incubated with radioactive ^14^C- or ^3^H-labeled fatty acids, such as palmitate. [^3^H]Palmitate is intracellularly converted to fatty-acyl-CoA and then incorporated into proteins. The degree of [^3^H]palmitate integration is then visualized by autoradiography (Bizzozero, 1995; Magee et al., 1995). However, such a procedure causes all endogenous proteins that undergo palmitoylation to incorporate the radiolabeled palmitate. Therefore, optimal labeling can only be achieved if the rate of synthesis and speed of palmitoylation turnover for the protein of interest are taken into account (Veit et al., 2008). In the final step, labeled proteins are enriched and analyzed using sodium dodecyl sulfate-polyacrylamide gel electrophoresis, exposed to X-ray films, and detected by fluorography.

In addition to detecting palmitoylation, radiolabeling is also utilized to monitor S-PALM turnover *in vitro*. The PULSE-CHASE labeling method has been successfully applied for decades. This method allows determination of the half-lives of the S-PALM of various proteins, including ankyrin (Staufenbiel, 1987), Gαi (Degtyarev et al., 1994), α2A-adrenergic receptors (Kennedy and Limbird, 1994), HTT (Sanders and Hayden, 2015), BACE1 (Bhattacharyya et al., 2016), H-Ras (Baker et al., 2003), and N-Ras (Magee et al., 1987). In this approach, cells are exposed to radioactively labeled palmitate for a short period of time (i.e., the PULSE phase), followed by a phase in which cells are incubated with unlabeled (non-radioactive) palmitate (i.e., the CHASE phase). During S-PALM turnover, proteins lose the radioactive palmitate, and a gradual decrease in the radioactive signal is observed. Although it is a very sensitive and effective method for the absolute quantification of protein palmitoylation, it has several limitations. Notably, special care is needed when handling radioactive materials. The procedure is also hazardous and time-consuming, requiring several weeks to months for autoradiography exposure. Finally, this method requires high levels of isotopically labeled fatty acid. The major limitation is that this approach can be utilized only for a single protein that is predicted to be palmitoylated. Furthermore, S-PALM stoichiometry (i.e., the ratio of palmitoylated protein and its unpalmitoylated form) cannot be determined. The determination of palmitoylation sites is possible but only in combination with mutations of predicted sites or in combination with limited enzymatic digestion and specific antibodies that target regions of interest. Radiolabeling has been replaced by other much more sensitive techniques.

### Metabolic Non-radioactive Labeling

An alternative to the radiolabeling approach utilizes metabolic labeling combined with click chemistry. This method was introduced by (Martin and Cravatt (2009) and showed great promise in the large-scale profiling of protein palmitoylation. The metabolic method involves labeling cells with a biorthogonal alkyne tag- or azide tag-containing analog of palmitic acid (Drisdel and Green, 2004; Roth et al., 2006; Kang et al., 2008). Different chemical reporters can be used in this process, such as Alk-16 or 17-ODYA (Charron et al., 2009a,b; Hannoush and Arenas-Ramirez, 2009), HDYOA (Yount et al., 2011), Alk-14 (Kostiuk et al., 2008), Az-15 (Charron et al., 2009a,b), and Az-14-CoA (Kostiuk et al., 2008). Chemical reporters and natural fatty acids (fatty acyl-CoA) have similar sizes and structures, thus allowing their recognition by cellular acyltransferases. Palmitic acid derivatives that are incorporated into proteins that comprise the azide/alkyne moiety are subsequently conjugated to reporter molecules, mainly fluorophores or biotin derivatives.

The method that was introduced by Martin and Cravatt (2009) utilizes the commercially available alkyne fatty acid analog 17-octadecynoic acid (17-ODYA) and click chemistry (Charron et al., 2009b). In the first step, 17-ODYA is fed into cells and actively incorporated into proteins that undergo palmitoylation. Next, 17-ODYA alkyne groups of modified proteins are conjugated with an azide-tagged reporter using a Cu(I)-catalyzed azide-alkyne cyclo addition reaction (CuAAC, click chemistry). The type of chemical reporter strictly depends on the downstream analysis. Generally, biotin or fluorescent rhodamine is used as a reporter molecule for the enrichment and detection of palmitoylated proteins using both mass spectrometry (MS) and other methods. The fluorescent reporters allow the robust in-gel visualization of S-PALM proteins, whereas the biotin-azide reporter is commonly used for streptavidin enrichment or Western blot. However, the visualization of palmitoylated proteins with the latter requires additional steps, such as transfer to a membrane and subsequent detection. However, both of them can lead to problems. Transfer efficiency can vary dramatically among proteins, depending on the ability of a protein to migrate out of the gel and its propensity to bind to the membrane. Additionally, immunodetection can generate a non-specific background. Importantly, however, this type of analysis can highlight endogenously biotinylated proteins. Currently, the biotin-azide reporter is mainly used for the enrichment of palmitoylated proteins and MS (Charron et al., 2009a; Martin and Cravatt, 2009; Yount et al., 2011).

Metabolic labeling with 17-ODYA and click chemistry coupled with MS allows the global profiling of palmitoylated proteins. Using this approach, over 300 S-PALM proteins have been identified in Jurkat T-cells, including SNAP23, RAP2B, calnexin, GNAQ, and flotillin 1. Similar to radiolabeling, the dynamics of palmitoylation turnover can be monitored with PULSE-CHASE analysis by fatty acid analogs (Zhang et al., 2010; Martin et al., 2011; Martin, 2013).

Differences in global palmitoyl dynamics under various conditions can be described by combining with the stable isotope labeling with amino acids in cell culture (SILAC) technique. In the standard SILAC approach, cells that express the tagged bait protein are fed with growth medium that contains the light isotope, and control cells are metabolically labeled with the heavy isotope or inversely (Wang and Huang, 2014). After metabolic labeling and cell lysis, equal amounts of light and heavy isotope-marked cell lysates are mixed and then purified using biotin-avidin affinity chromatography, followed by trypsin digestion and MS. Further analysis relies on measuring the ratio of light to heavy isotope-labeled forms for peptide pairs. Thus, comparisons of mass spectral peak intensities of peptide pairs allow the calculation of their relative abundance ratio, which is the basis for distinguishing specific proteins from non-specific background (Wang and Huang, 2014; Peng et al., 2016; Thinon et al., 2016). Metabolic labeling coupled with SILAC enables the identification of fatty-acylated proteins with high confidence by evaluating the signal-to-background ratio and comparisons of protein acylation levels. Using this approach, the stimulation of glutamate receptors was shown to accelerate palmitoylation turnover (Fukata and Fukata, 2010). Among the identified palmitoylated proteins that undergo dynamic palmitoylation are Ras-family GTPases, G proteins, membrane-associated guanylate kinase (MAGUK) proteins, and leucine-rich repeat and PDZ domain (LAP) proteins. Thus, this method allows the determination of context-dependent changes in protein S-PALM that are induced by specific cellular signaling pathways. Unfortunately, this approach cannot be used to study palmitoylation in large organisms. Moreover, 17-ODYA is a known potent cytochrome P450 hydroxylase inhibitor that plays an important role in the metabolism of fatty acids. However, its effect on palmitoyl metabolism in living systems is still unclear. Similar to radiolabeling, the relative quantification of protein palmitoylation remains challenging because unpalmitoylated proteins are not visible. This method is based on the metabolic labeling of cells with exogenous fatty acid analogs, and other fatty-acylated proteins (e.g., *N-*myristoylated proteins, *N*^𝜀^-myristoylated proteins, and GPI-anchored modified proteins) are also labeled (Peng et al., 2016). Additionally, care must be taken at each step of this approach to minimize the loss of palmitoyl groups.

### Click Chemistry-Proximity Ligation Assay

An extremely useful modification of the click chemistry method was its coupling with the proximity ligation assay (PLA). The latter was developed and first described by Fredriksson et al. (2002). The PLA is a relatively simple technique that is based on an immunocytochemical reaction that utilizes one pair of oligonucleotide-coupled antibodies that bind in close proximity (<40 nm) to different epitopes of protein or two interacting proteins. Following enzyme-mediated ligation of the added oligonucleotides, the obtained DNA circle is amplified, and the signal is then developed using fluorescently labeled oligonucleotides that are complementary to the replicated DNA. For many years, the PLA has been widely used for advanced and precise protein analysis (Gullberg et al., 2004; Söderberg et al., 2006). The principle of the click chemistry-PLA method has been reported (Gao and Hannoush, 2014). The authors showed that this approach can be successfully used to visualize cellular protein palmitoylation at the single protein level. In addition to imaging a specific palmitoylated cellular protein, this method can be applied to investigate the localization of this S-PALM protein using subcellular organelle markers. However, some limitations of this method should be considered. For example, it can only be used with fixed samples because of the toxicity of copper reagents that are needed for the click reaction. Another problem involves the fixation of cells with methanol because this causes the denaturation of many reporter proteins, such as green fluorescent protein (GFP) and red fluorescent protein (RFP). Therefore, this assay cannot be applied to cells that are transfected with plasmids that encode these proteins. Additionally, it is not always appropriate to use azide-tagged biotin to conjugate alkynyl palmitic acid because some tissues are characterized by high levels of endogenous biotin that can generate non-specific signals. To date, however, it is the only known method for the single-cell *in situ* imaging of S-PALM.

**FIGURE 7:**
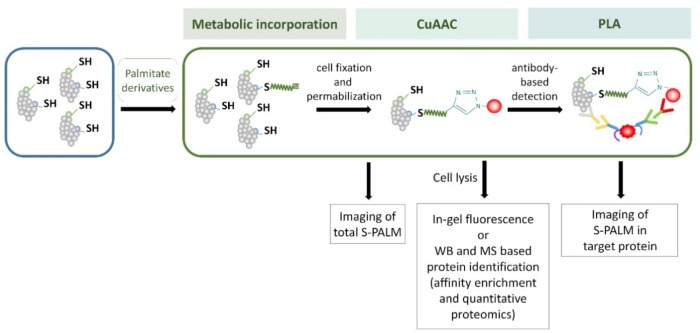
Schematic representation of the metabolic labeling workflow. S-PALM proteins can be studied using biorthogonal reactions. The cells are first incubated with palmitate derivatives (e.g., Alk-C16 and 17-ODYA; metabolic incorporation). Fixed and permeabilized cells are then processed for CuAAC (click chemistry) with either a fluorophore or affinity (biotin) tag. At this step, total S-PALM can be visualized by microscopy. Otherwise, after cell lysis, the level of S-PALM can be evaluated by in-gel fluorescence, Western blot (WB), or mass spectrometry (MS). For the single-cell *in situ* imaging of S-PALM in target protein, cells are subjected to the proximity ligation assay (PLA) using specific antibodies.

Metabolic labeling methods allow more sensitive and immediate detection compared with radioactive methods, and they are very useful for S-PALM protein determination and visualization in cell culture and cell lines. However, it is potentially difficult to apply these protocols to investigate protein *S*-acylation in tissue or cancer cells in which affected fatty-acid metabolism dramatically decreases the intake of exogenous long-chain fatty acids. Moreover, labeling methods require the application of modified palmitate acid analogs and prefer more stable S-PALM proteins than those with rapid palmitate turnover. The main steps of some of these metabolic labeling methods are shown in **Figure 7**.

## Biochemical Tools

**FIGURE 8:**
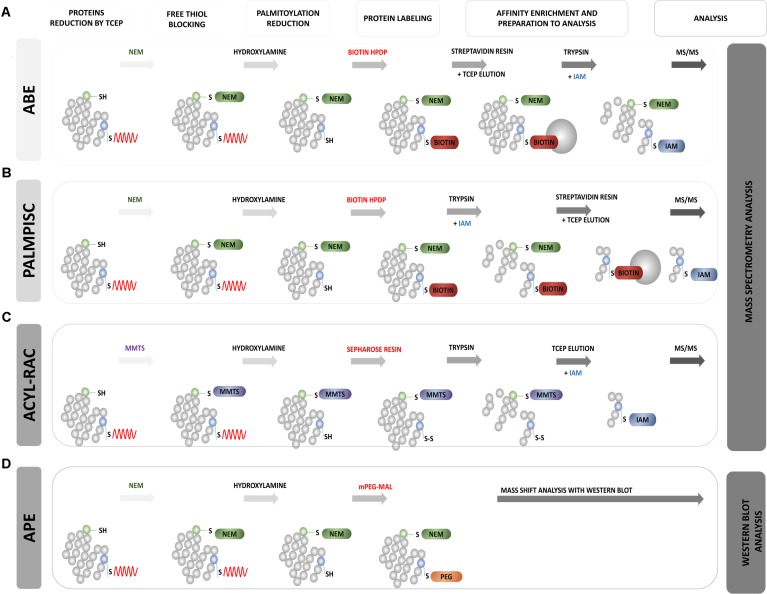
Schematic overview of biochemical assays for S-PALM identification. **(A)** Acyl Biotin Exchange (ABE). **(B)** Palmitoyl Protein Identification and Site Characterization (PalmPISC). **(C)** Resin Assisted Capture (AcylRAC). **(D)** Acyl-PEG Exchange (APE). The upper panel shows the particular stages of each assay. TCEP, Tris(2-carboxyethyl)phosphine; NEM, N-ethylmaleimide; IAM, iodoacetamide; MMTS, methyl methanethiosulfonate; mPEG-Mal, methoxy-PEG-maleimide; Biotin-HPDP, *N*-[6-(biotinamido)hexyl]-3′-(2′-pyridyldithio)propionamide; MS/MS, tandem mass spectrometry.

Biochemical approaches to palmitoylation analysis do not require metabolic labeling; instead, they take advantage of specific cleavage of the *S*-palmitoyl group by hydroxylamine. Drisdel and Green (2004) described a novel method that utilizes a radioactive alkylation reagent to introduce the palmitoyl group by chemical reactions *in vitro*. In this procedure, free thiol groups are first blocked with N-ethylmaleimide (NEM). Palmitoyl thioester linkages are then selectively liberated by hydroxylamine, and then newly exposed cysteinyl thiols are captured via [^3^H]N-ethylmaleimide. Labeled in this way, protein moieties are detected by autoradiography. Using this approach, The authors described the palmitoylation of three proteins that mediate proper functioning of the nervous system, namely 5-HT_3A_, SNAP-25, and PSD-95. They also showed that their method was much more sensitive than traditional metabolic labeling methods that utilize titrated palmitate. They also confirmed that this protocol can be applied to the analysis of *S*-acylated proteins in native cells, tissues, and biofluids. A simplified comparison of all of these described biochemical methods are shown in **Figure 8**.

### Acyl-Biotinyl Exchange Assay

Roth et al. (2006) based on the method of Drisdel and Green (2004), developed acyl-biotinyl exchange (ABE) chemistry and applied it to the detection of protein palmitoylation on a proteomic scale (Wan et al., 2007) (**Figure 8A**). This approach also involved blocking all free cysteine thiols with NEM and the specific removal of thioester-linked palmitoyl groups by hydroxylamine-induced hydrolysis. However, in the third step, non-radioactive labeling of the newly formed thiols with sulfhydryl-reactive biotin (biotin-HPDP) was used. The lack of specific palmitoyl antibodies in the past significantly limited studies of protein palmitoylation. Currently, the biotinylation of hydroxylamine-released thiol groups allows Western blot detection using streptavidin-horseradish peroxidase or the enrichment of biotinylated proteins with streptavidin resin for subsequent MS. In a standard control procedure, thioester-containing proteins are distinguished from non-specifically enriched proteins by comparing samples that are treated in parallel with and without hydroxylamine (Roth et al., 2006; Kang et al., 2008). The enriched protein fraction is analyzed using label-free MS. The application of this method to the yeast *Saccharomyces cerevisiae* protein fraction allowed the identification of 12 known and 35 novel palmitoylated proteins. This was the first demonstration of the large-scale profiling of protein palmitoylation (palmitoyl proteomics). The ABE protocol was further extended by Kang et al. (2008) to study *S*-acylated proteins from purified rat synaptosomes, embryonic rat neurons, and whole rat brain in three chemical steps. They identified 66 previously known neuronal palmitoyl proteins and more than 200 new *S*-palmitoylated candidates. The list of identified S-PALM proteins includes the scaffolding protein AKAP79/150, various ion channels, voltage-gated Na channels, and multiple transporters for neurotransmitter reuptake. Hemsley et al. (2013) showed that the ABE method can also be easily combined with isobaric tagging for the relative and absolute quantification (iTRAQ) method, which allows the quantitative analysis of modified proteins. Using this approach, they identified more than 580 S-PALM proteins from *Arabidopsis thaliana*. The ABE method was also applied to differentially analyzed changes in the S-PALM of proteins in postmortem brains of schizophrenia patients. They identified 219 palmitoylated proteins in human frontal cortex and observed a significant reduction of the palmitoylation of 17 proteins.

In the context of synaptic physiology, the ABE method was mainly used in combination with Western blot to detect single protein targets of S-PALM. Using this type of analysis, the S-PALM of important synaptic proteins was identified, such as LIM kinase, AMPA, and the NMDA NR2A and NR2B receptor subunits (Sato et al., 2009; Thomas and Huganir, 2013). This method was also applied to detect APP protein in a model of AD. Bhattacharyya et al. (2013) used two independent methods, metabolic labeling with Alk-16 and ABE, and found that two N-terminal cysteine residues of APP undergo S-PALM.

The advantage of the ABE approach is palmitoyl protein enrichment from complex samples through the specific binding of biotin to avidin-resin, followed by release of the protein from beads by the simple reduction of disulfide bonds. Both single targeted protein and total protein extracts can be analyzed using liquid chromatograph (LC)-MS. Thus, ABE allows the identification of protein S-PALM targets but does not provide information about the exact site of S-PALM.

### Palmitoyl Protein Identification and Site Characterization

A partial solution to the aforementioned problem was the development of the Palmitoyl Protein Identification and Site Characterization (PalmPISC) technique (Yang et al., 2010) (**Figure 8B**). Similar to ABE, PalmPISC involves the selective exchange of an S-PALM Cys residue to biotinylated cysteine. PalmPISC also involves the trypsin digestion of all proteins prior to avidin affinity chromatography. This approach leads to the enrichment of only biotinylated peptides that are originally palmitoylated. After disulfide bond reduction, these peptides are released from the affinity column and analyzed by MS. PalmPISC allows pinpointing S-PALM proteins and precise sites of S-PALM modification. This method has been successfully used to analyze palmitoylation in tumor cell lines. It was also able to identify 67 known and 331 novel S-PALM proteins, including AP2A2, ADAM10, AP2, and Ahnak, and precisely determine 25 known and 143 new S-PALM sites.

A variant of the PalmPISC technique was published, with minor changes in cysteine labeling (Collins et al., 2017). This method, called site-specific Acyl Biotin Exchange (ssABE), also relies on selectively switching the S-PALM Cys residue to biotinylated Cys. In the first step, free Cys thiols are blocked with iodoacetamide (IAA) instead of NEM, which is normally used in PalmPISC. The S-PALM Cys residues are then converted to biotin, and only the previously modified peptides are purified using streptavidin resin. Eluted peptides that contain free Cys are further analyzed by MS. Using this method, The authors identified 906 palmitoylation sites on 641 proteins from mouse forebrain homogenates. Notably, however, The authors did not explain the lack of use of a blocking agent, which may lead to false positives.

### Resin-Assisted Capture of *S*-Acylated Proteins

A simple alternative to ABE is the detection of *S*-acylated proteins by resin-assisted capture (Acyl-RAC) at the biotinylation site (Forrester et al., 2010, 2011) (**Figure 8C**). The Acyl-RAC method was initially applied to identify *S*-nitrosylation (SNO) sites in proteins. This assay is chemically analogous to the ABE method, but it replaces the biotin enrichment steps with direct conjugation with resin that contains thiol-reactive thiopyridine groups, which simplifies purification (Zhou et al., 2014). This method can be easily adapted to investigate other reversible cysteine modifications, such as *S*-glutathionylation (SSG), disulphide formation, and SNO, which differ in reducing agents (e.g., glutaredoxin, dithiothreitol, ascorbate, and hydroxylamine) (Guo et al., 2014). Similar to ABE, Acyl-RAC coupled with LC-MS/MS and isobaric labeling with iTRAQ reporter tags allowed the identification of new *S*-acylated substrates, such as the β-subunit of the protein translocating system (Sec61b). Acyl-RAC was applied by Forrester et al. (2011) to the *S*-acylation detection of endogenous and overexpressed H-Ras in a mammalian cell system. The specificity of this assay was demonstrated with C181/184S double mutants, which cannot be *S*-acylated and were not detected with the Acyl-RAC method.

### Acyl-PEG Exchange

New mass-shift labeling method, APE, was developed by Percher et al. (2016, 2017) (**Figure 8D**). This approach utilizes the hydroxylamine-sensitivity of thioesters and selective reactivity of Cys residues for site-specific alkylation with maleimide-functionalized polyethylene glycol reagents. APE induces a mass-shift on *S*-acylated proteins that can be directly verified by the Western blot analysis of target proteins with omitting the metabolic labeling or affinity enrichment of proteins (Percher et al., 2016). The authors tested this new method with several cell lines (HEK293T, NIH 3T3, and HeLa). They demonstrated the possibility of its application. They first showed that the endogenous interferon-induced transmembrane protein 3 (IFITM3), an important immune effector, is *S*-acylated, and its antiviral activity was directly correlated with the level of site-specific modifications of highly conserved Cys residues (Percher et al., 2016, 2017). Therefore, an important feature of the APE assay is its ability to disclose the ratio of *S*-acylated vs. unmodified proteins of multiple sites of *S*-fatty acylation, which is crucial for understanding the ways in which quantitative differences in the levels of *S*-acylation control protein function and associated cellular phenotypes. Although APE allows the characterization of relative levels of *S*-fatty acylated proteins and their isoforms, it does not reveal whether specific proteins are directly *S*-acylated or the dynamics of modifications at specific sites.

All of the aforementioned methods, despite the many advantages of protein palmitoylation analysis, have several limitations. They generally require multiple reactions and the purification of samples by triplicate precipitation between each reaction step, which can cause substantial sample loss. Moreover, the incomplete blockade of free Cys residues can result in false positives. False negatives can also occur because of insufficient thioester hydrolysis by hydroxylamine, inefficient biotin labeling (for ABE, APE, and PalmPISC), or undefined enrichment using various resins.

A summary of the main advantages and disadvantages of the aforementioned methods is presented in **Table 1**.

**Table 1:** Advantages and disadvantages of *S*-palmitoylation detection and identification methods.

METHOD	ADVANTAGES	DISADVANTAGES	EXAMPLES OF IDENTIFIED PROTEINS	REFERENCE
***METABOLIC LABELING***


*Metabolic radioactive labeling*	• Sensitive and effective • Allows monitoring S-PALM turnover	• Can be utilized only for a single protein • The S-PALM/unpalmitoylated protein ratio cannot be determined • Does not provide information on the exact site of S-PALM •Radioactive • Hazardous and time-consuming	Ankyrin, Gαi, α2A-adrenergic receptor, H-Ras, N-Ras, p21N-ras BACE1, HTT, GluK2, 5-HT_1A_, D_1_R, 5-HT_1B_, 5-HT_3A_, 5-HT_4_, and 5-HT_7_	Schmidt et al., 1979; Magee et al., 1987; Staufenbiel, 1987; Degtyarev et al., 1994; Kennedy and Limbird, 1994; Bizzozero, 1995; Magee et al., 1995; Jin et al., 1999; Baker et al., 2003; Papoucheva et al., 2004; Kvachnina et al., 2005, 2009; Ponimaskin et al., 2005; Renner et al., 2007; Veit et al., 2008; Copits and Swanson, 2013


*Metabolic non-radioactive labeling*	• Allows robust in-gel visualization • Allows global profiling of S-PALM proteins • Allows monitoring S-PALM turnover	• Highlights endogenously biotinylated proteins • Cannot be used to study palmitoylation in higher organisms •Only *in vitro* studies • Non-specific (other fatty-acylated proteins are labeled) • Does not provide information on the exact site of *S*-palmitoylation	APP, SNAP23, RAP2B, calnexin, D_2_R GNAQ, and flotillin 1	Drisdel and Green, 2004; Roth et al., 2006; Charron et al., 2009a,b; Hannoush and Arenas-Ramirez, 2009; Martin and Cravatt, 2009; Fukata and Fukata, 2010; Yount et al., 2011; Ebersole et al., 2015


*Click chemistry-proximity ligation assay*	• Allows visualization of single S-PALM protein • Provides information on S-PALM protein subcellular localization • Can be used to investigate colocalization of S-PALM protein with other proteins	•Can only be used on fixed samples • Cannot be applied to cells that are transfected with plasmids that encode tagging proteins (e.g., GFP) • Can generate non-specific signals from endogenous biotin • Does not provide information on the exact site of S-PALM	Wnt, Sonic Hedgehog, H-Ras,	Gao and Hannoush, 2014


***BIOCHEMICAL TOOLS***


*Acyl-biotin exchange assay*	• Provides large-scale profiling of protein S-PALM • Allows quantitative analysis of S-PALM proteins • Both individual proteins and total protein extracts can be analyzed	• Does not provide information on the exact site of S-PALM •Requires multiple reactions and purification of samples, which can cause substantial sample loss •Incomplete blockade of free Cys residues can result in false positives •Indirect method • Insufficient hydrolysis of thioester by hydroxylamine or inefficient biotin labeling may generate false negatives	LIM kinase, AMPA, NMDA NR2A and NR2B receptor subunits, 5HT_3A_, SNAP-25, PSD-95, APP, VGLUT1, small GTPase Ras, MBP, D_3_R, D_4_R	Wan et al., 2007; Kang et al., 2008; Sato et al., 2009; Emmer et al., 2011; Ho et al., 2011; Hemsley et al., 2013; George et al., 2015; Zhang and Kim, 2016; Zhang et al., 2016


*Palmitoyl Protein Identification and Site Characterization*	• Provides large-scale profiling of protein S-PALM • Allows quantitative analysis of modified proteins • Both single proteins and total protein extracts can be analyzed • Allows identification and S-PALM site characterization	•Requires multiple reactions and purification of samples, which can cause substantial sample loss •Incomplete blockade of free Cys residues can result in false positives •Indirect method • Insufficient thioester hydrolysis by hydroxylamine or inefficient biotin labeling may generate false negatives	PSD-95, SNAP 25, NOS, Fasn, PLP, NMDA, glutamate receptor, AMPA, Ddah1, Gja1, AP2A2, ADAM10, AP2, or Ahna	Yang et al., 2010; Collins et al., 2017


*Resin-assisted capture of S-acylated proteins*	• Provides large-scale profiling of protein S-PALM • Allows quantitative analysis of modified proteins •Allows for S-PALM site identification	•Requires multiple reactions and purification of samples, which can cause substantial sample loss •Incomplete blockade of free Cys residues can result in false positives •Indirect method	Gα_s_, Gα_11,_ H-ras, Uba1, SNAP23, Sec61b	Forrester et al., 2010, 2011


*Acyl-PEG exchange*	• Allows visualization of single S-PALM protein • Allows visualization of a number of S-PALM sites	•Requires multiple reactions and purification of samples, which can cause substantial sample loss •Incomplete blockade of free Cys residues can result in false positives •Indirect method	IFITM3,	Percher et al., 2016, 2017




## Concluding Remarks

*S*-palmitoylation is a frequent lipid modification that targets proteins to specific membrane compartments and has crucial regulatory functions. Over the last decade, significant progress has been made in the development of methods for the analysis of protein acylation. These new proteomic approaches provide insights into the importance of S-PALM to nerve cell function. This modification plays an important role in neuronal development and regulates the synaptic targeting of many proteins, thereby enabling efficient synaptic transmission. Accumulating evidence indicates that defects in palmitoylation are associated with a wide range of brain abnormalities, ranging from mental retardation to neurological disorders. Despite the methodological and technical advances that are discussed in this review, new strategies and analytical improvements are still needed to address existing limitations and challenges. For example, new methods are still needed to monitor dynamic S-PALM and explain its role in both normal physiology and disease. New label-free methods are also necessary to investigate palmitoylation *in vivo* in cells or tissues. This will certainly contribute to further advances in our knowledge and understanding of the complex mechanisms of functional synaptic modulation.

## Author Contributions

MZ-K, IF, AB-K, and JW discussed findings, analyzed literature, and wrote the manuscript.

## Conflict of Interest Statement

The authors declare that the research was conducted in the absence of any commercial or financial relationships that could be construed as a potential conflict of interest.
